# A Study of the Stability Mechanism of the Dispersed Particle Gel Three-Phase Foam Using the Interfacial Dilational Rheology Method

**DOI:** 10.3390/ma11050699

**Published:** 2018-04-28

**Authors:** Xue Yao, Ping Yi, Guang Zhao, Xin Sun, Caili Dai

**Affiliations:** 1 School of Petroleum Engineering, China University of Petroleum (East China), Qingdao 266580, China; dqyaoxue@163.com (X.Y.); upcsunxin@163.com (X.S.); 2 National Engineering Laboratory for Exploration and Development of Low Permeability Oil and Gas Field, Xi’an 710018, China; yip3_cq@petrochina.com.cn

**Keywords:** dispersed particle gel (DPG), stability mechanism, interfacial dilational rheology, microstructure, viscoelastic particles

## Abstract

The dispersed particle gel (DPG) three-phase foam is a novel profile control and flooding system. The stability mechanism of the DPG three-phase foam was studied using an interfacial dilational rheology method. The results show that the elastic modulus of the DPG three-phase foam is up to 14 mN/m, which is much higher than the traditional foam. The increase in interface elasticity produces significantly positive effects on foam stability. Emphasis is given to the influences of frequency, temperature, pressure, and concentration on the viscoelasticity and interfacial adsorption of DPG particles, which change the modules of the foam interface and have a significant effect on foam stability. In addition, the microstructure of the DPG three-phase foam was observed. A viscoelastic shell is formed by the aggregation of the DPG particles on the interface. The irreversible adsorption gives the interface high elasticity and mechanical strength. The electrostatic repulsion between particles increases the spacing between bubbles. The combined effects of these factors give the interface higher mechanical strength, slow down the film drainage, effectively prevent gas permeation, and significantly improve the foam stability.

## 1. Introduction

Nowadays, the foam system has attracted a lot of attention, and it has found an increasingly wide utilization in various fields, such as petroleum engineering, food engineering, and for the synthesis of new materials [1,2,3,4,5,6,7,8,9]. Foam has been extensively applied to foam flooding, foam profile, foam fracture acidizing, foam drainage gas recovery, and other engineering processes in the oil industry. However, foam is a thermodynamically unstable system and is transient under high reservoir temperature and pressure or external disturbance factors [1]. It is of great importance to enhance foam stability. There are three effects that cause foam to burst in reservoirs: liquid film drainage, disturbance rupture of the liquid membrane, and gas diffusion [2]. Polymers, polymer gels, and nanoparticles are added into foams to enhance foam stability. They do this by increasing foam viscosity or particle adsorption in order to inhibit gas diffusion and retard the rate of liquid drainage [3,4,5,6]. However, these additives have some drawbacks that cannot bear harsh reservoir conditions. For example, the viscosity reduction of the polymers due to shear degradation make the foam viscosity lower and decrease foam stability. The variation in the wettability of nanoparticles under high temperatures and high salinity makes it difficult for nanoparticles to adsorb on the gas liquid interface, resulting in an unstable foam system [7]. G. Zhao [8,9] found that foam stability can be enormously enhanced by adding the dispersed particle gel (DPG). The novel three-phase foam has a large foam volume and a long life in reservoir conditions. The DPG particles have high viscoelasticity, and they can both adsorb on the foam interface and increase the liquid viscosity. Currently, however, the study of the DPG three-phase foam is still in its infancy, and the stability mechanism is still controversial.

Foam stability is mainly determined by the properties of the liquid film, so most early research was based on the measurement of the equilibrium interfacial tension [10,11]. With the enrichment of surfactant types and the expansion of its application fields, the study of the dynamic properties of the interface received more attention, and the interfacial rheology research gradually became a common method with which to measure the stability of foam [12,13], especially interfacial elasticity [6,11,14,15]. It is found that there is a numerical relationship between the interfacial viscoelasticity and the thickness of the liquid film [14,15]. The interfacial dilational rheology method is widely used to study the interface properties of surfactant solutions, nanoparticle-foam, the surfactant-polymer composite system, emulsions, and oil/water interfaces [16,17,18,19,20,21,22,23]. Information on aggregation morphology, rearrangement and diffusion orientation, microscopic relaxation, and the interaction of molecules can be obtained by interfacial rheology [24,25,26,27,28,29,30,31,32].

In this study, a new three-phase foam system was prepared by adding DPG particles into surfactant solutions. The interfacial rheological behavior of this composite foam system in different conditions was investigated by a dilational, rheological method. The mechanism of the effect of the DPG on interfacial behavior was discussed, which provided the theoretical basis for the mechanism of viscoelastic solid particles to stabilize foam.

## 2. Experimental Section

### 2.1. Materials

**Figure 1:**
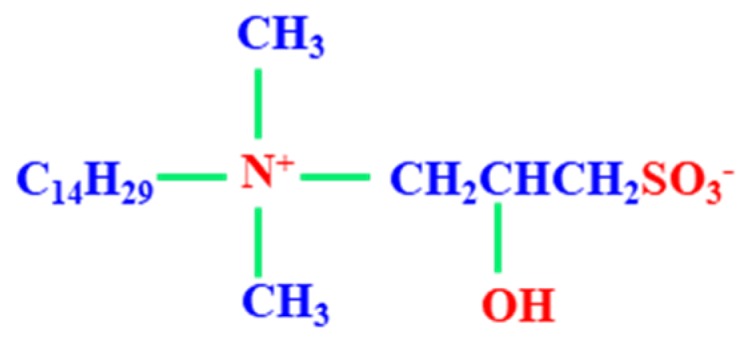
Chemical structure of THSB.

Tetradecyl hydroxyl sulfobetaine (THSB) used as foaming agent was provided by Nuosong Company, Limited, Shanghai, China. Chemical structure of THSB is shown in Figure 1. Partial hydrolysis polyacrylamide (HPAM) was provided by Oil Service Company, Langfang, China and had a molecular weight of 9.6 × 10^6^ (g·mol^−1^) and a hydrolysis degree of 3.62%. The phenolic resin crosslinker was provided by Shengli Oilfield Shengli Chemical Company, Limited, Dongying, China. The purity of nitrogen was more than 99%, and this was provided by the Tianyuan Company, Qingdao, China. All the stock solutions were prepared using simulated water with a salinity of 800 mg·L^−1^.

### 2.2. Preparation of the DPG Three-Phase Foam

**Figure 2:**
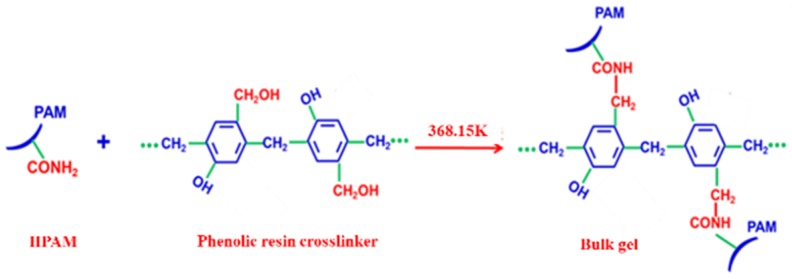
Reaction equation for the formation of bulk gel.

**Figure 3:**
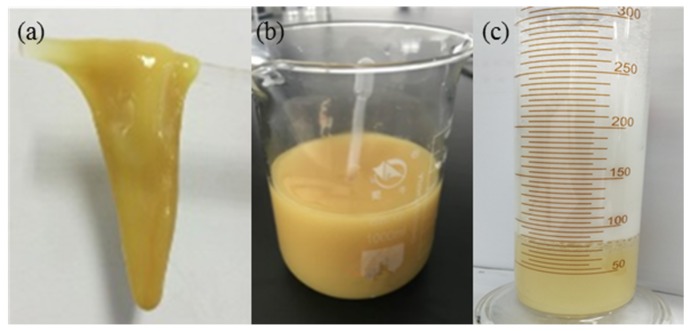
Pictures of bulk gel, DPG particles and DPG three-phase foam. (**a**) Bulk gel; (**b**) DPG particles; (**c**) DPG 3-phase foam.

The mixture of 0.3% HPAM and 0.9% phenolic resin crosslinker was placed into an oven for 6 h at 368.15 K until a bulk gel was formed. The reaction equation is shown in Figure 2. Then, the bulk gel was added to the JM-85 colloid mill rotating at 3000 rpm for 10 min at 298.15 K. The obtained pale yellow solution was the DPG solution with a concentration of 0.3%. The concentration of DPG solution is defined as the concentration of HPAM used in the preparation process. The DPG particles are mainly spherical with a size distribution between 1 and 3 μm, and the Zeta potential is from −28.6 to −36.2 mV; both were measured using a Particle Size-Zeta Potential Analyzer (Brookhaven NanoBrook Omni, New York, NY, USA). The viscosity of DPG particles is 5.2 mPa·s, measured by a viscometer (Brookfield DV−2 Pro, Middleboro, MA, USA) with a shear rate of 73 s^−1^. The DPG particles, THSB, and water were prepared into a mixture of different concentration ratios. Nitrogen was injected into the mixture to generate the three-phase foam. Pictures of bulk gel, DPG particles, and DPG three-phase foam are shown in Figure 3.

### 2.3. Interfacial Dilational Rheology

**Figure 4:**
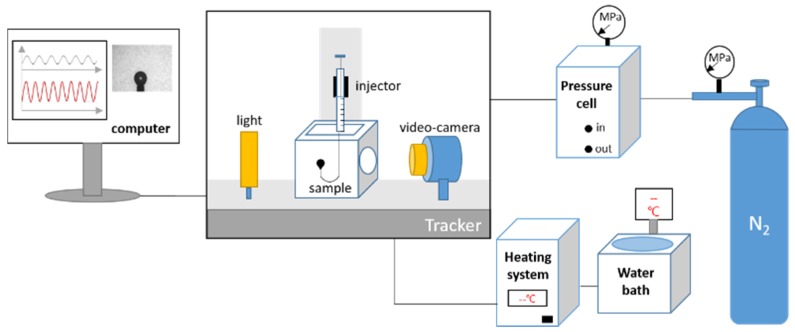
Schematic diagram of the interface rheometer.

Interfacial dilational rheology was performed using the interfacial tension meter/rheometer (Tracker-H) provided by TECLIS Company, Lyon, France. A schematic diagram of the device is shown in Figure 4. A bubble was formed on the end of a U-type needle immersed in the solution, and the profile of the floating bubble was transmitted to the computer application by a camera. The bubble volume controller made the floating bubble oscillate. The surface tension and the interfacial dilatation modulus of the nitrogen/water interface was calculated using a computer application. In the absence of special instructions, the experiment was carried out under the following conditions: (1) Temperature: 298.15 K; (2) Pressure: 101,325 Pa; (3) Vibration frequency: 0.1 s^−1^; and (4) Amplitude: 10% of the initial volume.

When the area of the interface sinusoidally fluctuates, molecules are continuously adsorbed and desorbed on the interface membrane, and the interfacial tension gives a sinusoidal response. The interfacial dilational modulus (*E*) is defined as follows,

E=dσ/(dA/A)=dσ/dlnA

in which *σ* is the gas/liquid interfacial tension and *A* is the bubble surface area.

The interfacial dilational modulus is the comprehensive characterization of the resistance to deformation ability and the repair ability of the deformation of the interface. The higher the value of the dilational modulus, the higher the film strength, and the stronger the self-repair ability.

For viscoelastic interfaces, the interfacial dilational modulus is composed of the elastic modulus and the viscous modulus,

E=|E|cosθ+i|E|sinθ

in which |E|cosθ represents dilational elasticity, |E|sinθ represents dilational viscosity, and *θ* is the phase angle, representing the proportion of the elastic modulus and the viscous modulus.

## 3. Results and Discussion

### 3.1. Evaluation of Foam Stability

**Figure 5:**
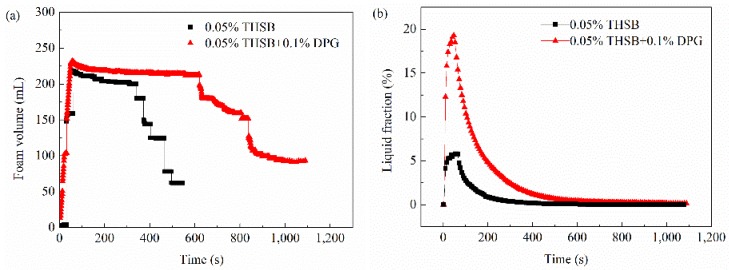
Foam volume and liquid fraction over time. (**a**) Foam volume and (**b**) liquid fraction in foam.

Lots of work [9] was done during the early stages of the stability evaluation of DPG three-phase foam. Therefore, the half-life time and drainage of the traditional aqueous foam and DPG three-phase foam at 363.15 K were specifically investigated using a foam scanner (TECLIS Company, Lyon, France). The experimental results are shown in Figure 5. In Figure 5a, the foam volume of the DPG three-phase foam stabilized for a while and then began to decrease until 650 s. However, the foam volume of the traditional aqueous foam began to dramatically decrease at 370 s. The half-life time of the DPG three-phase foam is 900 s, which is 2.2 times longer than the traditional aqueous foam. In Figure 5b, the liquid carrying capacity of the DPG three-phase foam is up to 20%, which is four times higher than the traditional aqueous foam. The time of liquid drainage was lengthened from 200 to 480 s, and the foam stability was significantly improved by adding DPG particles.

It is worth noting that the DPG three-phase foam was still maintained for about 400 s after the liquid fraction was close to zero. This is closely related to the property of the thin film and interface of foam. Therefore, interfacial rheology of foam was studied to reveal the mechanism of stability.

### 3.2. Interfacial Rheology and Factors Influencing Foam Stability

#### 3.2.1. Influence of the DPG Concentration

**Figure 6:**
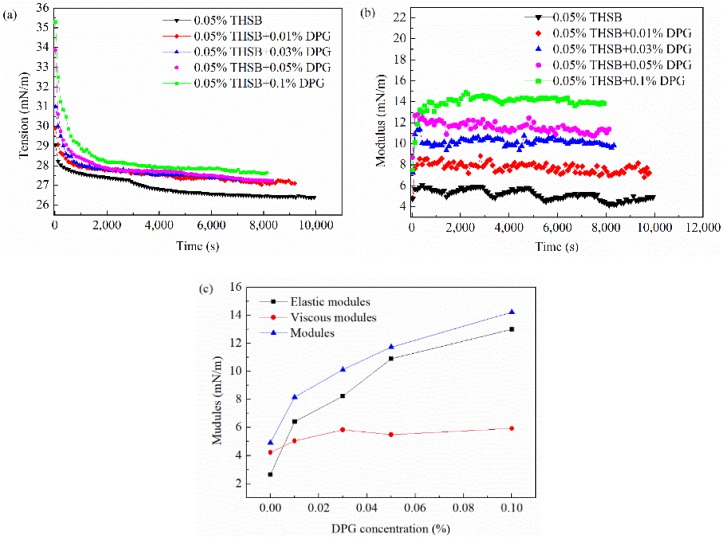
Interfacial tension and modulus at different DPG concentrations. (**a**) Dynamic interfacial tension; (**b**) dynamic interfacial dilational modulus; and (**c**) equilibrium interfacial modulus, elastic modulus, and viscous modulus.

The interfacial tension and modulus of the traditional aqueous foam (0.05% THSB) and the DPG three-phase foam at different DPG concentrations are shown in Figure 6. The interfacial tension of the DPG three-phase foam is slightly higher than that of the traditional foam. With an increase of DPG concentration, the dilational modulus significantly increases. In Figure 6c, the increase of the viscous modulus is slight, whereas the elastic modulus dramatically increases. Thus, the increase of the dilational modulus is mainly attributed to the increase of the elastic modulus. The elastic modulus derives from the energy change caused by the deviation from the equilibrium state after the perturbation of the interface molecules, which is closely related to the intermolecular interaction. The results show that the DPG three-phase foam has better mechanical strength, and the interface has better resistance to deformation.

In the traditional aqueous foam, the interfacial tension gradually decreases with an increase of surfactant molecules adsorbing on the interface, and equilibrium is obtained after saturated adsorption. The dilational modulus fluctuates with time, and the foam interface is unstable. The fast relaxation process of surfactant molecules includes molecular migration between the bulk and interface and molecules transporting on the interface. This process timely repairs the interfacial tension gradient caused by deformation, making the interface elastic modulus small and causing it to continuously fluctuate. In the DPG three-phase foam, the interfacial tension increases after DPG particles and surfactant molecules interact and aggregate on the interface because of the hydrophobic effect of the DPG particles. With an increase of the DPG concentration, the number of DPG particles on the interface increases, the interfacial tension continues to rise, and the increasing amplitude gradually decreases. An increase of surface tension will have an adverse effect on the foaming performance of the foam, but it has no decisive influence on the foam stability. DPG particles are viscoelastic macromolecules, and the interfacial tension of the foam film has a long equilibrium time because of the slow movement of DPG particles. When the interfacial area changes, its relaxation time is longer. There is not enough time to restore the tension gradient, and this leads to a larger tension gradient, which makes the interfacial dilational modulus higher. The adsorption of DPG particles with surfactant molecules increases the interfacial elasticity, makes the foam film thickness maintain a uniform state when it is disturbed, and improves the stability and strength of the foam film.

#### 3.2.2. Influence of the Surfactant Concentration

**Figure 7:**
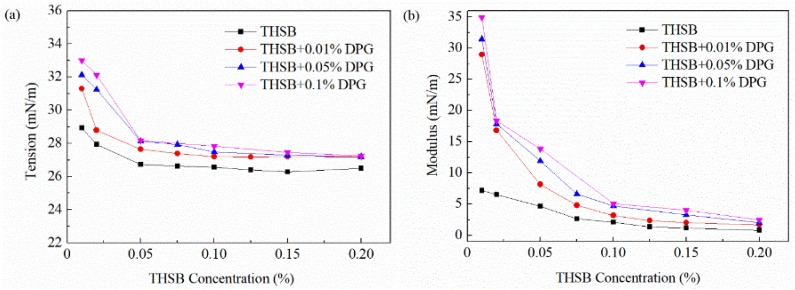
Equilibrium interfacial tension and modulus at different surfactant concentration. (**a**) Equilibrium interfacial tension and (**b**) equilibrium interfacial dilational modulus.

The equilibrium interfacial tension and modulus of the traditional aqueous foam and the DPG three-phase foam at different surfactant concentrations are shown in Figure 7. In the traditional aqueous foam, with an increase of surfactant concentration, the number of surfactant molecules adsorbed on the interface increases and the interfacial tension gradually decreases until the interface adsorption is saturated and reaches a plateau. At a high surfactant concentration, surfactant molecules in the liquid phase quickly transfer to the interface with the dilation of the bubble, and the interfacial tension quickly returns to the original state. Thus, the thinned liquid is not repaired, and the dilational modulus is low and the stability is poor. In the DPG three-phase foam, surfactant molecules associate with DPG particles and jointly adsorb on the interface. A large number of DPG particles aggregating on the interface at a low surfactant concentration leads to the dramatic improvement of the dilational modulus. At high surfactant concentrations, surfactant molecules begin to form free micelles, which largely distribute in the body phase and the interface. The decreasing number of DPG particles on the interface results in a lower modulus and a decrease in foam stability.

#### 3.2.3. Influence of Oscillation Frequency

**Figure 8:**
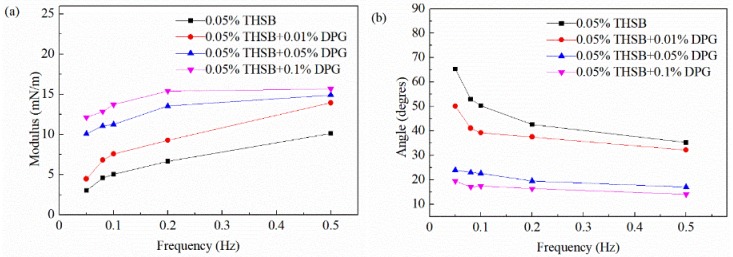
Equilibrium interfacial modulus and phase angle at different oscillation frequencies. (**a**) Equilibrium interfacial modulus and (**b**) equilibrium phase angle.

The oscillation frequency is an important factor affecting the dilational modulus. The equilibrium interfacial modulus and phase angle of the traditional aqueous foam and the DPG three-phase foam in the 0.05–0.5 Hz frequency range are shown in Figure 8. At a low DPG concentration, with an increase of the oscillation frequency, the dilational modulus increases and the phase angle decreases. At a high DPG concentration, the number of DPG particles at the interface increases, and the frequency has less influence on the interfacial modulus.

The migration rate of surfactant molecules is fast. At a low oscillation frequency, molecules have enough time to repair the interfacial tension gradient using the Marangoni effect and bulk and interfacial molecular mass transfer. The greater the oscillation frequency, the faster the deformation rate of the interface, resulting in a shorter time for surfactant molecules to repair the tension gradient and a greater elastic modulus. As the relaxation process of active molecules is fast and the action time is short, the influence of the viscous part on the modulus is less than the elastic part, resulting in a decrease of the phase angle.

The DPG is a macromolecule polymer, and, thus its relaxation process is very slow. When the interfacial area changes, there is almost no mass transfer and desorption during the experimental period. The change of frequency has little effect on the interfacial tension gradient, and the elastic modulus of the interface remains stable. The surface film has higher elasticity and a better ability to restore the deformation.

#### 3.2.4. Influence of Temperature

**Figure 9:**
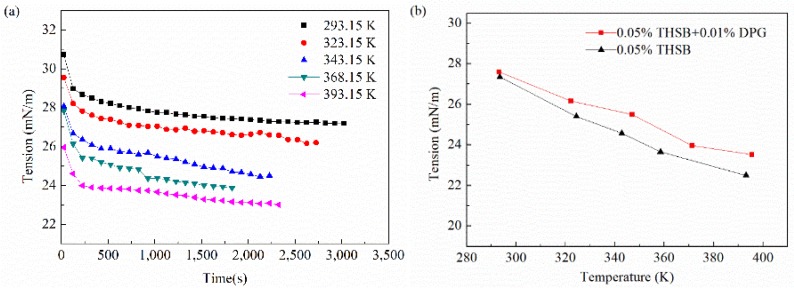

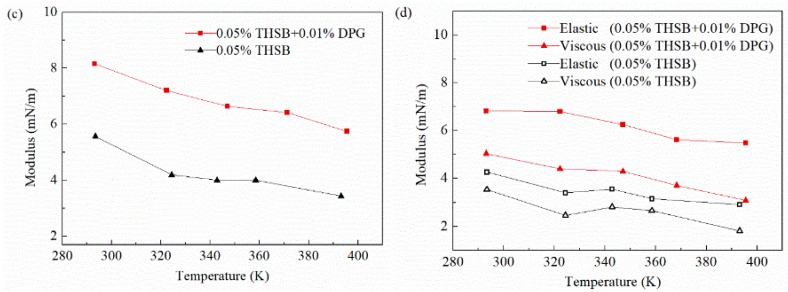
Interfacial tension and modulus under different temperatures. (**a**) Dynamic interfacial tension (0.05% THSB + 0.01% DPG), (**b**) equilibrium interfacial tension, (**c**) equilibrium interfacial modulus, and (**d**) equilibrium interfacial viscous modulus and elastic modulus.

The interfacial tension and modulus of the traditional aqueous foam and the DPG three-phase foam at different temperatures are shown in Figure 9. The higher the temperature, the faster the interface reaches equilibrium, the lower the equilibrium interfacial tension, and the smaller the viscous modulus and the elastic modulus. The interfacial modulus of the DPG three-phase foam at a high temperature is obviously higher than that of the traditional aqueous foam.

High temperature accelerates the thermal motion of molecules, which is conducive to the activity of molecular aggregation at the interface and rapidly promotes a decrease in interfacial tension. However, when the temperature is too high, the gas-liquid mass transfer gets faster. The interfacial tension gradient caused by deformation decreases; the interfacial modulus and the interfacial elasticity decrease, and it is easier for the gas to pass through the liquid membrane. Also, as the temperature rises, the evaporation of liquid gets faster, and the speed of film drainage is quicker, which causes the instability of the foam. At high temperatures, DPG particles still maintain good molecular activity and form effective adsorption on the interface to maintain the high elasticity of the film. The heat transfer resistance of the liquid film is increased by the elastic membrane structure. Therefore, the liquid film drainage and gas transmission are less affected by temperature, and the foam has better stability.

#### 3.2.5. Influence of Pressure

**Figure 10:**
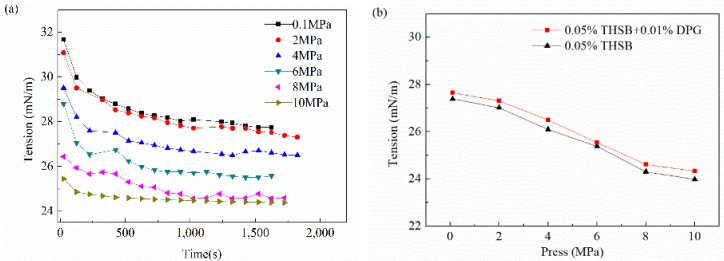

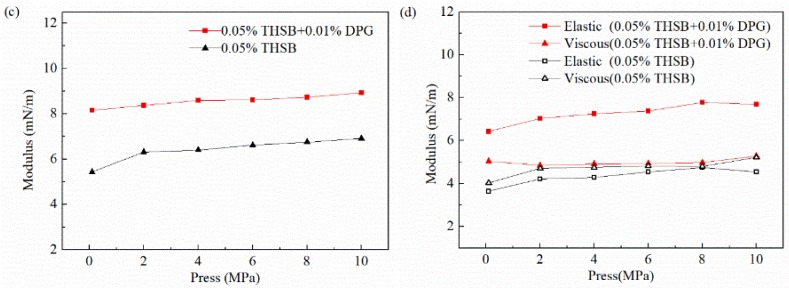
Interfacial tension and modulus under different pressures. (**a**) Dynamic interfacial tension, (**b**) equilibrium interfacial tension, (**c**) equilibrium dilational modulus, and (**d**) equilibrium viscous modulus and elastic modulus.

The interfacial tension and modulus of the traditional aqueous foam and the DPG three-phase foam at different pressures are shown in Figure 10. When the pressure rises, the balance time of the foam interface is basically unchanged, and the interfacial tension is reduced. As the pressure continues to increase, the interfacial tension slowly decreases. The interfacial tension reaches the minimum value when the pressure increases to 8 MPa, which is called the minimum equilibrium interfacial tension pressure. The equilibrium interfacial modulus slightly rises with an increase of pressure and finally reaches equilibrium. The interfacial modulus of the DPG three-phase foam at different pressures is obviously higher than that of the traditional aqueous foam.

Pressure has little effect on the molecular diffusion rate. When the pressure rises, the foam interface is compressed, and the number of DPG particles and active molecules increase and form a closer arrangement on the interface, resulting in a decrease of interfacial tension and an increase of interfacial modulus. This plays an important role in enhancing the liquid membrane strength. As the pressure continues to rise, the interfacial tension and modulus reach a plateau after saturated adsorption of molecules. Under high pressure, the interfacial film is more elastic and the foam is more stable.

### 3.3. Analysis of the Foam Stabilization Mechanism

**Figure 11:**
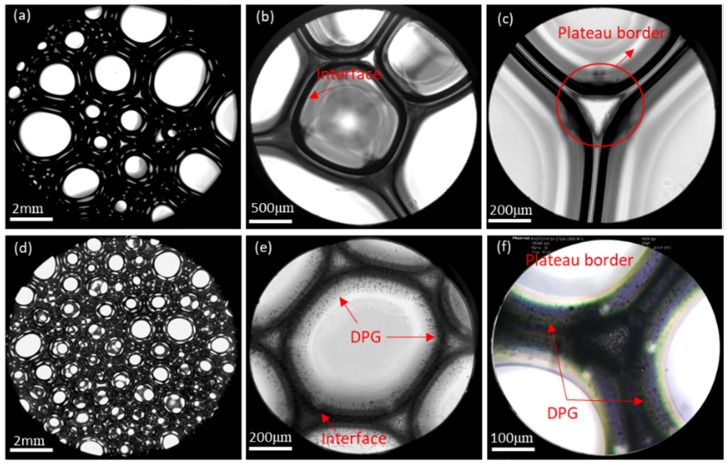
Microstructure of the traditional aqueous foam and the DPG three-phase foam. (**a**,**b**) The traditional aqueous foam at different levels of magnification, (**c**) the plateau border of the traditional aqueous foam, (**d**) the DPG three-phase foam, (**e**) DPG particle adsorption at the gas-liquid interface and non-adsorption in the solution, and (**f**) the plateau border of the DPG three-phase foam.

The microstructure of the traditional foam and the DPG three-phase foam was observed using a Leica DMi8 C inverted microscope system, as shown in Figure 11. The traditional foam is loosely arranged with different bubble sizes (Figure 11a). The liquid film is thin with little distance between the interfaces, and can be easily ruptured (Figure 11a,c). In the DPG three-phase foam, the bubbles are dense and uniform (Figure 11d), and a large number of particles gather in the liquid phase and on the interface, resulting in a significant increase in the thickness of the liquid film and the spacing between the interfaces (Figure 11e,f).

DPG particles stabilize the foam in two ways. The first way is through the formation of adsorption layer on the liquid film. The second way is through the dispersal in the liquid phase. DPG particles associate with surfactant molecules by physical adsorption and gather on the foam interface. The DPG particles that adsorbed on the interface are tightly arranged, forming a dense and stable elastic membrane structure. The thickness of the liquid film obviously increases, and the liquid drainage channel is blocked. This can be seen as steric stabilization. Besides, the strong elastic shell forms a skeleton of the foam to wrap up the gas, reducing the contact area between the bubbles, preventing the coalescence of bubbles, and weakening the permeation of the liquid film and gas. The elastic film has good mechanical strength. It is not easy to break up the foam by external disturbances, which results in a more stable foam system. Moreover, the excess DPG particles dispersing uniformly in the bulk phase form an orderly structure. When the bubbles are close to each other, solid particles will hinder liquid drainage. Thus, the stability of the foam is improved.

**Figure 12:**
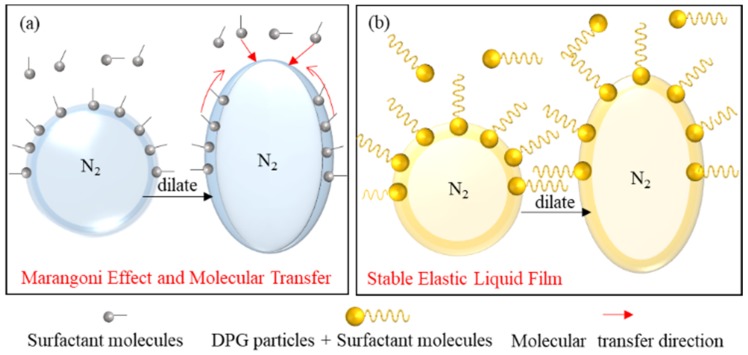
Foam stability mechanism of the surfactant and DPG particles. (**a**) Traditional aqueous foam and (**b**) DPG three-phase foam.

Furthermore, the traditional foam is mainly maintained by the Marangoni effect under a low surfactant concentration. When the surfactant concentration is high, the rapid migration of molecules from the liquid phase to the interface will reduce the Marangoni effect, and the thinned liquid film cannot be repaired, resulting in the poor stability of the foam. DPG particles are polymer particles with viscoelasticity and have solid particle characteristics. The particles form a stable elastic membrane structure on the gas-liquid interface. The relaxation process of DPG macromolecules is very slow when external disturbances occur. There is no mass transfer and desorption of DPG particles in the body phase and on the interface during the experimental period, and therefore the thickness of the foam film remains uniform. This irreversible adsorption gives the interface high elasticity and mechanical strength, which is beneficial to the stability of the foam. The schematic diagram of the mechanism of foam stabilization is shown in Figure 12.

In addition, as DPG particles and THSB molecules are negatively charged, the electrostatic repulsion between particles increases the spacing between bubbles, and this weakens the stratification and coalescence of the foam film.

The combined effect of all factors above gives the interface higher mechanical strength, slows down the liquid film drainage rate, effectively prevents gas permeation, and significantly improves the foam stability.

## 4. Conclusions

The stability mechanism of the DPG three-phase foam in different conditions was experimentally studied using an interfacial dilational rheology method. The results show that frequency, temperature, pressure, and concentration influence the viscoelasticity and interfacial adsorption of DPG, which change the dilational modules of the foam interface and have a significant effect on foam stability. The stability mechanism of the DPG three-phase foam is as follows. DPG particles and surfactant molecules are closely arranged on the foam interface by physical adsorption, forming a viscoelastic stable membrane structure. This strong elastic shell forms the skeleton of the foam to wrap up the gas, preventing the coalescence of bubbles. The unadsorbed DPG particles are evenly dispersed in the body phase to slow down the liquid film drainage. The relaxation process of DPG particles is very slow. Under an external disturbance, there is almost no mass transfer and desorption of particles on the interface. The thickness of the foam film remains uniform, and the liquid film has higher mechanical strength and restoration ability. The electrostatic repulsion between the negative interfaces causes the spacing between bubbles to increase. The combined effect of all factors above gives the interface higher mechanical strength, slows down the liquid film drainage rate, effectively prevents gas permeation, and significantly improves the foam stability.
